# A gadoxetic acid-enhanced MRI-based model using LI-RADS v2018 features for preoperatively predicting Ki-67 expression in hepatocellular carcinoma

**DOI:** 10.1186/s12880-024-01204-9

**Published:** 2024-01-25

**Authors:** Yingying Liang, Fan Xu, Qiuju Mou, Zihua Wang, Chuyin Xiao, Tingwen Zhou, Nianru Zhang, Jing Yang, Hongzhen Wu

**Affiliations:** 1 Department of Radiology, Guangzhou First People’s Hospital, School of Medicine, South China University of Technology, 1Panfu Road, Guangzhou, Guangdong Province 510180 China; 2 Department of Radiology, Guangzhou Red Cross Hospital, Medical College, Jinan University, 396 Tongfu Road, Guangzhou, Guangdong Province 510220 China; 3 Department of Pathology, Guangzhou First People’s Hospital, School of Medicine, South China University of Technology, 1Panfu Road, Guangzhou, Guangdong Province 510180 China; 4 Department of Radiology, Foshan Hospital of Traditional Chinese Medicine, Foshan, Guangdong Province 528000 China

**Keywords:** Hepatocellular carcinoma, Magnetic resonance imaging, Ki-67, Diagnosis

## Abstract

**Purpose:**

To construct a gadoxetic acid-enhanced MRI (EOB-MRI) -based multivariable model to predict Ki-67 expression levels in hepatocellular carcinoma (HCC) using LI-RADS v2018 imaging features.

**Methods:**

A total of 121 patients with HCC who underwent EOB-MRI were enrolled in this study. The patients were divided into three groups according to Ki-67 cut-offs: Ki-67 ≥ 20% (*n* = 86) vs. Ki-67 < 20% (*n* = 35); Ki-67 ≥ 30% (*n* = 73) vs. Ki-67 < 30% (*n* = 48); Ki-67 ≥ 50% (*n* = 45) vs. Ki-67 < 50% (*n* = 76). MRI features were analyzed to be associated with high Ki-67 expression using logistic regression to construct multivariable models. The performance characteristic of the models for the prediction of high Ki-67 expression was assessed using receiver operating characteristic curves.

**Results:**

The presence of mosaic architecture (*p* = 0.045), the presence of infiltrative appearance (*p* = 0.039), and the absence of targetoid hepatobiliary phase (HBP, *p* = 0.035) were independent differential factors for the prediction of high Ki-67 status (≥ 50% vs. < 50%) in HCC patients, while no features could predict high Ki-67 status with thresholds of 20% (≥ 20% vs. < 20%) and 30% (≥ 30% vs. < 30%) (*p* > 0.05). Four models were constructed including model A (mosaic architecture and infiltrated appearance), model B (mosaic architecture and targetoid HBP), model C (infiltrated appearance and targetoid HBP), and model D (mosaic architecture, infiltrated appearance and targetoid HBP). The model D yielded better diagnostic performance than the model C (0.776 vs. 0.669, *p* = 0.002), but a comparable AUC than model A (0.776 vs. 0.781, *p* = 0.855) and model B (0.776 vs. 0.746, *p* = 0.076).

**Conclusions:**

Mosaic architecture, infiltrated appearance and targetoid HBP were sensitive imaging features for predicting Ki-67 index ≥ 50% and EOB-MRI model based on LI-RADS v2018 features may be an effective imaging approach for the risk stratification of patients with HCC before surgery.

## Introduction

Hepatocellular carcinoma (HCC) is the most common primary liver cancer and ranks second in cancer-related mortality worldwide [1]. High tumor recurrence and metastasis, which occurs in approximately 60%–70% of patients within 5 years, remains a major concern in HCC treatment [2–5]. Patients with the same types of tumors receiving the same treatments at the same doses may have different outcomes due to differences in the proliferative activities of tumors.

Ki-67 is a nuclear antigen that is only expressed during the cell proliferation phase and has a short half-life [6–8]. As such, it is an effective biomarker to predict tumor cell division and proliferative activity, which is believed to be associated with the therapeutic effects and prognoses of malignant tumors in clinical practice [6–9]. The optimal cut-off value of Ki-67 to guide the clinical management of patients with HCC remains undetermined, although previous studies have shown that high Ki-67 expression is associated with tumor differentiation, lymph node metastasis, and poor prognoses [6, 10]. Currently, Ki-67 can only be evaluated by surgery or biopsy histopathology, which are invasive and may cause infection, intra-abdominal bleeding, and tumor spread [11]. In addition, puncture biopsy has a high rate of misdiagnosis due to sampling error. Therefore, there is an urgent need for a non-invasive method to predict the optimal cut-off value of Ki-67 for the risk stratification of patients with HCC.

Gadoxetic acid-enhanced magnetic resonance imaging (EOB-MRI) can play an essential role in the diagnosis, staging, and surveillance of HCC. To standardize the interpretation of features in imaging reports and promote communication between different HCC-related disciplines, the Liver Imaging Reporting and Data System (LI-RADS) was introduced in 2016 by the American College of Radiology [12]. Recent meta-analysis reported that LI-RADS showed moderate sensitivity of 62–67% and high specificity of 91–93% for diagnosing HCC [13, 14]. This high specificity at the cost of sensitivity was designed for the prevention of misallocation of liver transplants. Emerging pieces of studies suggested that EOB-MRI has high clinical value for preoperatively predicting Ki-67 expression in HCC, however, the clinical promotion has been limited because the commercial software is required to transform the results [15–18].

Thus, this study aimed to explore the correlation between EOB-MRI LI-RADS v2018 features and different Ki-67 expression levels, and construct a multivariable model based on EOB-MRI using LI-RADS v2018 features for preoperative prediction of Ki-67 expression in patients with HCC.

## Materials and methods

### Patients

This retrospective single-center study was approved by the Institutional Review Board with waived requirement for informed consent (Ethical Board Approval Number: “K-2022–004-01”). From January 2017 to April 2023, all patients with pathologically confirmed HCC in our hospital were included in this study. 136 patients were excluded from the study; 67 were due to incomplete pathological data, 36 had previous treatment for HCC, 27 were due to incomplete MRI sequence, and the other 6 were due to poor quality of MRI images caused by respiratory motion artifacts.

**Fig. 1:**
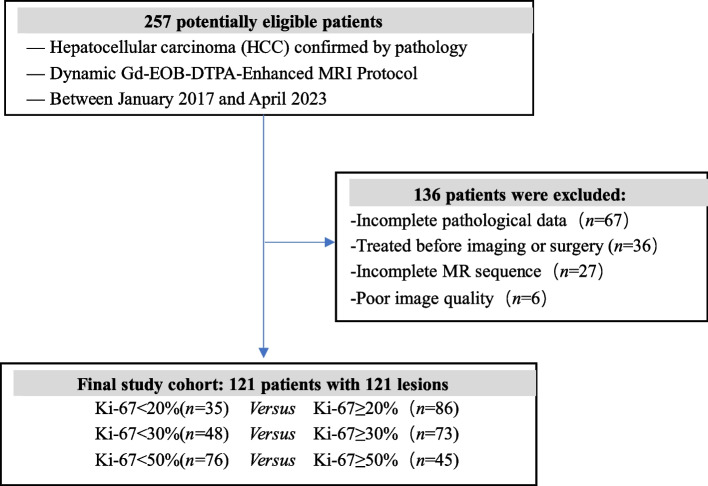
Flow chart of the study population selection

Lastly, 121 patients with HCC were enrolled in this study. Figure 1 shows a flow chart of the study population. According to different thresholds of 20%, 30% and 50% of Ki-67, HCC lesions were divided into three groups: Ki-67 ≥ 20% (*n* = 86) vs. Ki-67 < 20% (*n* = 35); Ki-67 ≥ 30% (*n* = 73) vs. Ki-67 < 30% (*n* = 48); Ki-67 ≥ 50% (*n* = 45) vs. Ki-67 < 50% (*n* = 76) [6, 19, 20].

### Clinical and laboratory data

All clinical and laboratory data of the patient were retrieved and collected from the clinical case system. The characteristics including age, gender, etiology of the underlying liver disease, Child-pugh score, the levels of alpha-fetoprotein (AFP), alanine aminotransferase (ALT), serum total bilirubin (STB), plasma albumin (PA), and platelet levels were selected for distinguishing HCC with different thresholds of Ki-67.

### MRI examinations

MRI was obtained using a 3.0T MRI scanner (Siemens Magnetom Verio). Pre-contrast MRI sequences included T1-weighted imaging, T2-weighted imaging, and diffusion-weighted imaging (DWI). EOB-DTPA (Primovist, Bayer Schering Pharma, Germany) was injected at a dose of 0.025 mmol/kg at 2 mL/s for contrast-enhanced MRI including hepatic arterial phase (AP), portal venous phase (PVP), transitional phase (TP), and hepatobiliary phase (HBP) images.

### Image analysis

All images were evaluated by two abdominal radiologists (with 10 and 17 years of experience, respectively) independently, who were blinded to the final pathological diagnosis. Inter-observer agreement was assessed, and any discrepancies were resolved by consensus as the reference standard.

LI-RADS v2018, including major (non-rim arterial phase hyperenhancement [APHE], non-peripheral washout, enhancing capsule and threshold growth), ancillary (favoring HCC in particular, and favoring malignancy, not HCC in particular), and LR-M imaging criteria (targetoid appearance and non-targetoid LR-M features) were used to evaluate all lesions [21]. The threshold growth was not applicable because only one MRI examination per patient was evaluated in the analysis. Other imaging features, including intratumoral arteries (continuous enhancement of arterial vessels in the tumor during the arterial phase which attenuated in the portal phase and later phase), satellite nodules(presence of nodules ≤2 cm in diameter and within 2 cm of primary tumor), peritumoral enhancement(irregular enhancement outside the tumor margin in arterial phase which attenuated in portal phase and later phase), lymph node metastasis (the short axis of a lymph node was greater than 10 mm or central necrosis was found on MRI), portal and hepatic vein tumor thrombus (unequivocal enhancing soft tissue in portal and hepatic veins), and ascites, were also evaluated which has been reported in our previous study [22]. LI-RADS category of each lesion was assigned by the same two abdominal radiologists. In addition, the largest tumor was evaluated in patients with multiple tumors.

### Histopathological examination

The pathological reports of all included patients with HCC were retrospectively reviewed. The Ki-67 proliferation index was evaluated according to the normal immunohistochemical process and evaluated blindly by two experienced pathologists blindly.

### Statistical analysis

Continuous variables were compared using student’s *t* test or the Mann–Whitney U test and categorical variables were compared using χ^2^ test or Fisher’s exact test. Kappa (k) statistics were used to evaluate the agreement for MRI features (poor, 0.00–0.20; fair, 0.21–0.40; moderate, 0.41–0.60; substantial, 0.61–0.80; and excellent, 0.81–1.00). Data from the most experienced radiologist were used for analyses.

Univariate and multivariate logistic regression analyses were performed to identify the independent risk factors for predicting Ki-67 expression. Univariate predictors with *p* < 0.1 were used in the multivariate regression analysis.

Subsequently, different logistic regression models were built based on MRI features. For assessment of the discriminative abilities of the parameters and models, receiver operating characteristic (ROC) curves were constructed, and the areas under the ROC curves (AUC) were computed. The DeLong test was performed to compare AUCs of the prediction models. Two-sided *p* < 0.05 were considered statistically significant. All statistical tests were performed using SPSS (version 19.0, SPSS, Chicago, IL, USA).

## Results

### Clinical characteristics

The clinical characteristics of HCC patients are shown in Table 1. One hundred twenty-one patients (mean age, 56.73 ± 13.1 years), including 97 men and 24 women were analyzed. Child–Pugh classes A and B were found in 88.4% (107/121) and 11.6% (14/121) of patients, respectively. The most common etiology of HCC was HBV infection (96.7%), followed by chronic alcohol ingestion (2.5%) and primary biliary cirrhosis (0.8%). Of 121 HCC nodules, 14 (11.6%) and 76 (62.8%) were categorized as LR-4 and LR-5, respectively, whereas 6 (4.9%) and 25 (20.7%) were categorized as LR-M and LR-TIV, respectively. Larger tumor diameters (≥ 5 cm) were more prevalent in HCC with Ki-67 indexes < 20% (*p* = 0.837), ≥ 30% (*p* = 0.179), and ≥ 50% (*p* = 0.070) (Table 2).

**Table 1:** Patient characteristics

Index	20%			30%			50%		
	**Ki-67 < 20%**	**Ki-67 ≥ 20%**	***P***	**Ki-67 < 30%**	**Ki-67 ≥ 30%**	***P***	**Ki-67 < 50%**	**Ki-67 ≥ 50%**	***P***
**Sex**									
Male	29(82.9%)	68(79.1%)	0.636	39(81.3%)	58(79.5%)	0.808	62(81.6%)	35(77.8%)	0.612
Female	6(17.1%)	18(20.9%)		9(18.7%)	15(20.5%)		14(18.4%)	10(22.2%)	
**Age** (Mean ± SD)	59.57 ± 14.4	55.57 ± 12.4	0.129	57.71 ± 14.4	56.08 ± 12.2	0.507	57.38 ± 13.8	55.62 ± 11.8	0.478
**LI-RADS**									
4	5(14.3%)	9(10.5%)	0.121	7(14.6%)	7(9.6%)	0.015	11(14.5%)	3(6.7%)	0.036
5	26(74.3%)	50(58.1%)		36(75.0%)	40(54.8%)		52(68.4%)	24(53.3%)	
M	0(0.0%)	6(7.0%)		0(0.0%)	6(8.2%)		2(2.6%)	4(8.9%)	
TIV	4(11.4%)	21(24.4%)		5(10.4%)	20(27.4%)		11(14.5%)	14(31.1%)	
**AFP** (≥ 400)									
Positive	25(71.4%)	57(66.3%)	0.583	35(72.9%)	47(64.4%)	0.326	54(71.1%)	28(62.2%)	0.315
Negative	10(28.6%)	29(33.7%)		13(27.1%)	26(35.6%)		22(28.9%)	17(37.8%)	
**Etiology**									
Alcohol	2(5.7%)	1(1.2%)	0.284	2(4.2%)	1(1.4%)	0.454	2(2.6%)	1(2.2%)	0.733
HBV	33(94.3%)	84(97.6%)		46(95.8%)	71(97.2%)		73(96.1%)	44(97.8%)	
PBC	0(0.0%)	1(1.2%)		0(0.0%)	1(1.4%)		1(1.3%)	0(0.0%)	
**Child–pugh**									
A	32(91.4%)	75(87.2%)	0.511	45(93.8%)	62(84.9%)	0.138	72(94.7%)	35(77.8%)	0.005
B	3(8.6%)	11(12.8%)		3(6.3%)	11(15.1%)		4(5.3%)	10(22.2%)	
**ALT**(U/L)	47.51 ± 54.7	64.14 ± 75.8	0.241	44.38 ± 48.4	69.16 ± 80.7	0.058	53.24 ± 67.8	69.60 ± 74.5	0.219
**STB** (umol/L) (Mean ± SD)	28.85 ± 18.3	23.18 ± 16.8	0.103	26.30 ± 17.9	23.84 ± 17.1	0.448	24.32 ± 15.7	25.66 ± 20.0	0.682
**PA**(g/L) (Mean ± SD)	37.81 ± 3.9	37.21 ± 6.5	0.615	38.17 ± 3.8	36.87 ± 6.9	0.234	37.84 ± 4.2	36.61 ± 7.9	0.266
**Platelet** (× 10^9^/L) (Mean ± SD)	170.0 ± 123.2	174.98 ± 78.0	0.795	167.45 ± 110.4	177.55 ± 79.5	0.562	171.95 ± 95.6	176.33 ± 87.7	0.803

*Abbreviations*: *AFP* alpha-fetoprotein, *ALT* alanine aminotransferase, *HBV* Hepatitis B, *M* mean, *PA* plasma albumin, *PBC* primary biliary cirrhosis, *SD* standard deviation, *STB* serum total bilirubin

**Table 2:** The MRI imaging features of patients with different cut-off value of Ki-67

**Characteristics**		**20%**				**30%**				**50%**			**k Value**^a^
		**Ki-67 < 20%**	**Ki-67 ≥ 20%**	***P***		**Ki-67 < 30%**	**Ki-67 ≥ 30%**	***P***		**Ki-67 < 50%**	**Ki-67 ≥ 50%**	***P***	
Diameter (≥ 5 cm)	N	18(51.4%)	46(53.5%)	0.837	N	29(60.4%)	35(47.9%)	0.179	N	45(59.2%)	19(42.2%)	0.070	
	P	17(48.6%)	40(46.5%)		P	19(39.6%)	38(52.1%)		P	31(40.8%)	26(57.8%)		
**Major features of HCC**													
Non-rim APHE	N	5(14.3%)	20(23.3%)	0.269	N	7(14.6%)	18(24.7%)	0.181	N	17(22.4%)	8(17.8%)	0.547	0.948 (0.877–1.000)
	P	30(85.7%)	66(76.7%)		P	41(85.4%)	55(75.3%)		P	59(77.6%)	37(82.2%)		
Nonperipheral washout	N	3(8.6%)	20(23.3%)	0.062	N	6(12.5%)	17(23.3%)	0.139	N	16(21.1%)	7(15.6%)	0.456	0.868 (0.755–0.981)
	P	32(91.4%)	66(76.7%)		P	42(87.5%)	56(76.7%)		P	60(78.9%)	38(84.4%)		
Capsule enhancement	N	6(17.1%)	24(27.9%)	0.214	N	10(20.8%)	20(27.4%)	0.413	N	19(25.0%)	11(24.4%)	0.945	0.934 (0.861–1.000)
	P	29(82.9%)	62(72.1%)		P	38(79.2%)	53(72.6%)		P	57(75.0%)	34(75.6%)		
**Ancillary features (AF) that favor HCC over non-HCC malignancies**													
Non-enhanced capsule	N	32(91.4%)	74(86.0%)	0.415	N	40(83.3%)	66(90.4%)	0.248	N	65(85.5%)	41(91.1%)	0.368	0.792 (0.616–0.968)
	P	3(8.6%)	12(14.0%)		P	8(16.7%)	7(9.6%)		P	11(14.5%)	4(8.9%)		
Nodule in nodule	N	17(48.6%)	29(33.7%)	0.127	N	23(47.9%)	23(31.5%)	0.069	N	39(51.3%)	7(15.6%)	< 0.001	0.878 (0.790–0.966)
	P	18(51.4%)	57(66.3%)		P	25(52.1%)	50(68.5%)		P	37(48.7%)	38(84.4%)		
Mosaic architecture	N	18(51.4%)	30(34.9%)	0.092	N	24(50.0%)	24(32.9%)	0.060	N	43(56.6%)	5(11.1%)	< 0.001	0.898 (0.818–0.977)
	P	17(48.6%)	56(65.1%)		P	24(50.0%)	49(67.1%)		P	33(43.4%)	40(88.9%)		
Blood products in mass	N	25(71.4%)	61(70.9%)	0.956	N	35(72.9%)	51(69.9%)	0.717	N	59(77.6%)	27(60.0%)	0.039	0.980 (0.941–1.000)
	P	10(28.6%)	25(29.1%)		P	13(27.1%)	22(30.1%)		P	17(22.4%)	18(40.0%)		
Fat in mass, more than adjacent liver	N	30(85.7%)	71(82.6%)	0.672	N	38(79.2%)	63(86.3%)	0.301	N	63(82.9%)	38(84.4%)	0.824	0.908 (0.806–1.000)
	P	5(14.3%)	15(17.4%)		P	10(20.8%)	10(13.7%)		P	13(17.1%)	7(15.6%)		
**AF favoring malignancies in general, not HCC in particular**													
Mild to moderate T2 hyperintensity	N	0(0.0%)	3(3.5%)	0.263	N	0(0.0%)	3(4.1%)	0.155	N	0(0.0%)	3(6.7%)	0.023	0.853 (0.569–1.000)
	P	35(100.0%)	83(96.5%)		P	48(100.0%)	70(95.9%)		P	76(100.0%)	42(93.3%)		
Restricted diffusion	N	0(0.0%)	2(2.3%)	0.363	N	0(0.0%)	2(2.7%)	0.248	N	1(1.3%)	1(2.2%)	0.705	0.954 (0.890–1.000)
	P	35(100.0%)	84(97.7%)		P	48(100.0%)	71(97.3%)		P	75(98.7%)	44(97.8%)		
Corona enhancement	N	34(97.1%)	77(89.5%)	0.168	N	47(97.9%)	64(87.7%)	0.045	N	73(96.1%)	38(84.4%)	0.025	0.760 (0.559–0.961)
	P	1(2.9%)	9(10.5%)		P	1(2.1%)	9(12.3%)		P	3(3.9%)	7(15.6%)		
Fat sparing in a solid mass	N	26(74.3%)	65(75.6%)	0.881	N	36(75.0%)	55(75.3%)	0.966	N	55(72.4%)	36(80.0%)	0.347	0.890 (0.814–1.000)
	P	9(25.7%)	21(24.4%)		P	12(25.0%)	18(24.7%)		P	21(27.6%)	9(20.0%)		
Iron sparing in a solid mass	N	35(28.9%)	85(98.8%)	0.522	N	48(100.0%)	72(98.6%)	0.415	N	76(100.0%)	44(97.8%)	0.192	1.000 (1.000–1.000)
	P	0(0.0%)	1(1.2%)		P	0(0.0%)	1(1.4%)		P	0(0.0%)	1(2.2%)		
Transitional phase hypointensity	N	0(0.0%)	5(5.8%)	0.145	N	1(2.1%)	4(5.5%)	0.359	N	2(2.6%)	3(6.7%)	0.281	0.905 (0.720–1.000)
	P	35(100.0%)	81(94.2%)		P	47(97.9%)	69(94.5%)		P	74(97.4%)	42(93.3%)		
HBP hypointensity	N	0(0.0%)	4(4.7%)	0.194	N	1(2.1%)	3(4.1%)	0.542	N	2(2.6%)	2(4.4%)	0.590	0.853 (0.569–1.000)
	P	35(100.0%)	82(95.3%)		P	47(97.9%)	70(95.9%)		P	74(97.4%)	43(95.6%)		
**LR-M features**													
Rim APHE	N	35(100.0%)	74(86.0%)	0.020	N	48(100.0%)	61(83.6%)	0.003	N	71(93.4%)	38(84.4%)	0.110	0.783 (0.578–0.987)
	P	0(0.0%)	12(14.0%)		P	0(0.0%)	12(16.4%)		P	5(6.6%)	7(15.6%)		
Peripheral “washout”	N	34(97.1%)	81(94.2%)	0.497	N	47(97.9%)	68(93.2%)	0.237	N	73(96.1%)	42(93.3%)	0.505	0.792 (0.512–1.000)
	P	1(2.9%)	5(5.8%)		P	1(2.1%)	5(6.8%)		P	3(3.9%)	3(6.7%)		
Delayed central enhancement	N	35(100.0%)	81(94.2%)	0.145	N	48(100.0%)	68(93.2%)	0.064	N	74(97.4%)	42(93.3%)	0.281	0.885 (0.661–1.000)
	P	0(0.0%)	5(5.8%)		P	0(0.0%)	5(6.8%)		P	2(2.6%)	3(6.7%)		
Targetoid restriction	N	35(100.0%)	74(86.0%)	0.020	N	46(95.8%)	63(86.3%)	0.086	N	69(90.8%)	40(88.9%)	0.735	0.914 (0.796–1.000)
	P	0(0.0%)	12(14.0%)		P	2(4.2%)	10(13.7%)		P	7(9.2%)	5(11.1%)		
Targetoid TP	N	31(88.6%)	73(84.9%)	0.597	N	40(83.3%)	64(87.7%)	0.502	N	63(82.9%)	41(91.1%)	0.209	0.869 (0.744–0.995)
	P	4(11.4%)	13(15.1%)		P	8(16.7%)	9(12.3%)		P	13(17.1%)	4(8.9%)		
Targetoid HBP	N	23(65.7%)	57(66.3%)	0.953	N	30(62.5%)	50(68.5%)	0.496	N	46(60.5%)	34(75.6%)	0.091	0.909 (0.832–0.987)
	P	12(34.3%)	29(33.7%)		P	18(37.5%)	23(31.5%)		P	30(39.5%)	11(24.4%)		
Infiltrative appearance	N	13(37.1%)	32(37.2%)	0.995	N	21(43.8%)	24(32.9%)	0.226	N	35(46.1%)	10(22.2%)	0.009	0.911 (0.835–0.987)
	P	22(62.9%)	54(62.8%)		P	27(56.2%)	49(67.1%)		P	41(53.9%)	35(77.8%)		
Marked diffusion restriction	N	6(17.1%)	23(26.7%)	0.262	N	10(20.8%)	19(26.0%)	0.513	N	16(21.1%)	13(28.9%)	0.329	0.796 (0.406–1.000)
	P	29(82.9%)	63(73.3%)		P	38(79.2%)	54(74.0%)		P	60(78.9%)	32(71.1%)		
Necrosis or severe ischemia	N	16(45.7%)	38(44.2%)	0.878	N	23(47.9%)	31(42.5%)	0.555	N	37(48.7%)	17(37.8%)	0.243	0.967 (0.921–1.000)
	P	19(54.3%)	48(55.8%)		P	25(52.1%)	42(57.5%)		P	39(51.3%)	28(62.2%)		
**Other imaging features**													
Intratumoral artery	N	20(57.1%)	58(67.4%)	0.283	N	28(58.3%)	50(68.5%)	0.253	N	51(67.1%)	27(60.0%)	0.430	0.888 (0.802–0.975)
	P	15(42.9%)	28(32.6%)		P	20(41.7%)	23(31.5%)		P	25(32.9%)	18(40.0%)		
Satellite nodules	N	26(74.3%)	52(60.5%)	0.150	N	36(75.0%)	42(57.5%)	0.050	N	52(68.4%)	26(57.8%)	0.237	0.907 (0.828–0.987)
	P	9(25.7%)	34(39.5%)		P	12(25.0%)	31(42.5%)		P	24(31.6%)	19(42.2%)		
Portal and hepatic vein tumor thrombus	N	31(88.6%)	66(76.7%)	0.139	N	43(89.6%)	54(74.0%)	0.035	N	65(85.5%)	32(71.1%)	0.055	0.974 (0.922–1.000)
	P	4(11.4%)	20(23.3%)		P	5(10.4%)	19(26.0%)		P	11(14.5%)	13(28.9%)		
Peritumoral enhancement	N	35(100.0%)	75(87.2%)	0.026	N	47(97.9%)	63(86.3%)	0.030	N	72(94.7%)	38(84.4%)	0.057	0.948 (0.846–1.000)
	P	0(0.0%)	11(12.8%)		P	1(2.1%)	10(13.7%)		P	4(5.3%)	7(15.6%)		
Lymph node metastasis	N	34(97.1%)	82(95.3%)	0.653	N	47(97.9%)	69(94.5%)	0.359	N	74(97.4%)	42(93.3%)	0.281	0.885 (0.661–1.000)
	P	1(2.9%)	4(4.7%)		P	1(2.1%)	4(5.5%)		P	2(2.6%)	3(6.7%)		
Ascites	N	32(91.4%)	73(84.9%)	0.335	N	40(83.3%)	65(89.0%)	0.365	N	66(86.8%)	39(86.7%)	0.978	0.963 (0.891–1.000)
	P	3(8.6%)	13(15.1%)		P	8(16.7%)	8(11.0%)		P	10(13.2%)	6(13.3%)		

*Abbreviations*: *APHE* arterial phase hyperenhancement, *HBP* hepatobiliary phase, *N* negative, *P* positive, *TP* transitional phase

^a^The interobserver agreement for each feature is described with Kappa (k) statistics

### Interobserver agreement

The interobserver agreement was substantial for nonenhanced capsule, corona enhancement, rim APHE, peripheral washout, and marked diffusion restriction [k = 0.760–0.796] and excellent for all major features of HCC [k = 0.868–0.948], AF that favor HCC over non-HCC malignancies except for nonenhanced capsule [k = 0.878–0.980], AF favoring malignancies in general, not HCC in particular except for corona enhancement [k = 0.853–1.000], LR-M features except for rim APHE, peripheral “washout”, and marked diffusion restriction [k = 0.869–0.967] and all other imaging features [k = 0.885–0.974] (Table 2).

### Univariate and multivariate analysis of differential factors between different Ki-67 cut-offs

The relationship between EOB-MRI features and the different Ki-67 cut-offs are presented in Tables 2 and 3.

**Table 3:** Multivariate analysis with logistic regression in the MRI imaging features

Characteristic	Multivariate analysis		
	B	*P*	OR (95%CI)
**20% of Ki-67 cutoff**			
Nonperipheral washout	-0.794	0.285	0.452 (0.105–1.938)
Mosaic architecture	0.796	0.077	2.216 (0.918–5.350)
Rim APHE	19.08	0.998	1.93 × 10^8^ (0)
Targetoid restriction	19.439	0.998	2.77 × 10^8^ (0)
Peritumoral enhancement	18.844	0.998	1.53 × 10^8^ (0)
**30% of Ki-67 cutoff**			
Nodule in nodule	-0.269	0.734	0.764 (0.162–3.599)
Mosaic architecture	0.897	0.251	2.453 (0.531–11.339)
Corona enhancement	0.826	0.505	2.285 (0.201–25.908)
Rim APHE	20.436	0.998	7.5 × 10^8^ (0)
Delayed central enhancement	19.197	0.999	2.17 × 10^8^ (0)
Targetoid restriction	0.082	0.935	1.086 (0.148–7.979)
Satellite nodules	0.207	0.680	1.23 (0.459–3.294)
Portal and hepatic vein tumor thrombus	0.657	0.307	1.93 (0.547–6.805)
Peritumoral enhancement	0.703	0.569	2.02 (0.179–22.736)
**50% of Ki-67 cutoff**			
Size (≥ 5 cm)	-0.852	0.280	0.427(0.091–2.003)
Nodule in nodule	0.862	0.365	2.367(0.367–15.28)
Mosaic architecture	1.706	0.045^*^	5.507(1.036–29.271)
Blood products in mass	0.149	0.810	1.16(0.346–3.895)
Mild to moderate T2 hyperintensity	-21.537	0.999	0(0)
Corona enhancement	2.195	0.078	8.983(0.785–102.793)
Targetoid HBP	-1.304	0.035^*^	0.271(0.081–0.915)
Infiltrated appearance	1.141	0.039^*^	3.129(1.058–9.258)
Portal and hepatic vein tumor thrombus	0.108	0.862	1.114(0.330–3.764)
Peritumoral enhancement	0.309	0.776	1.362(0.163–11.381)

*Abbreviations*: *APHE* arterial phase hyperenhancement, *B* regression coefficients, *CI* confidence interval, *HBP* hepatobiliary phase, *OR* odds ratio^*^p < 0.05

^*^p < 0.05

For Ki-67 ≥ 20%, univariate analysis suggested that absence of nonperipheral washout (*p* = 0.062), presence of mosaic architecture (*p* = 0.092), presence of rim APHE (*p* = 0.020), presence of targetoid restriction (*p* = 0.020), and presence of peritumoral enhancement (*p* = 0.026)were potential differential factors for the prediction of HCC with Ki-67 ≥ 20%. Subsequently, multivariate logistic analysis was conducted on these potential differential factors; no factors were significantly different.

**Fig. 2:**
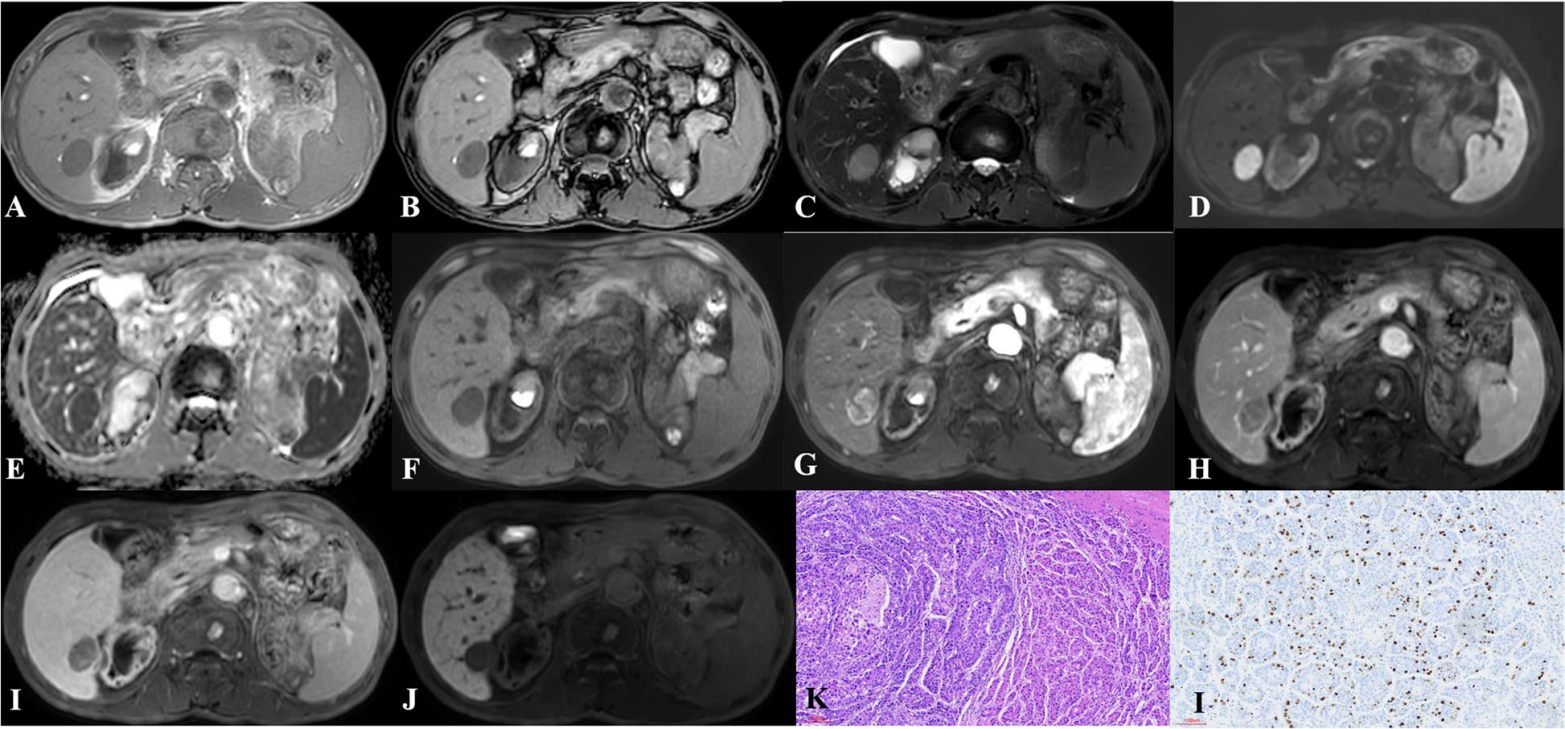
Hepatocellular carcinoma in the right lobe of liver showed hypointense on T1-weighted imaging (**A**, **B**), hyperintense on T2-weighted imaging (**C**), hyperintense on DWI (**D**) while hypointense on apparent diffusion coefficient map (**E**), hypointense on precontrast T1-weighted imaging (**F**), arterial phase hyperenhancement (APHE) on arterial phase (**G**), washout and enhancing capsule on portal venous phase (**H**) and transitional phase (**I**), and apparent hypointense without targetoid appearence on hepatobiliary phase (**J**). **H** Pathology revealing hepatocellular carcinoma (HE × 10). **I** Immunohistochemistry showing high proliferative activity of tumor cells with approximately 10% Ki-67 expression (× 10)

For Ki-67 ≥ 30%, univariate analysis suggested that the presence of nodule-in-nodule architecture (*p* = 0.069), presence of mosaic architecture (*p* = 0.060), presence of coronal enhancement (*p* = 0.045), presence of rim APHE (*p* = 0.003), presence of delayed central enhancement (*p* = 0.064), presence of targetoid restriction (*p* = 0.086), presence of satellite nodules (*p* = 0.050), presence of portal and hepatic vein tumor thrombus (*p* = 0.035), and presence of peritumoral enhancement (*p* = 0.030) were potential differential factors for the prediction of HCC with Ki-67 ≥ 30%. Subsequently, multivariate logistic analysis was conducted on these potential differential factors; no factors were significantly different (Fig. 2).

**Fig. 3:**
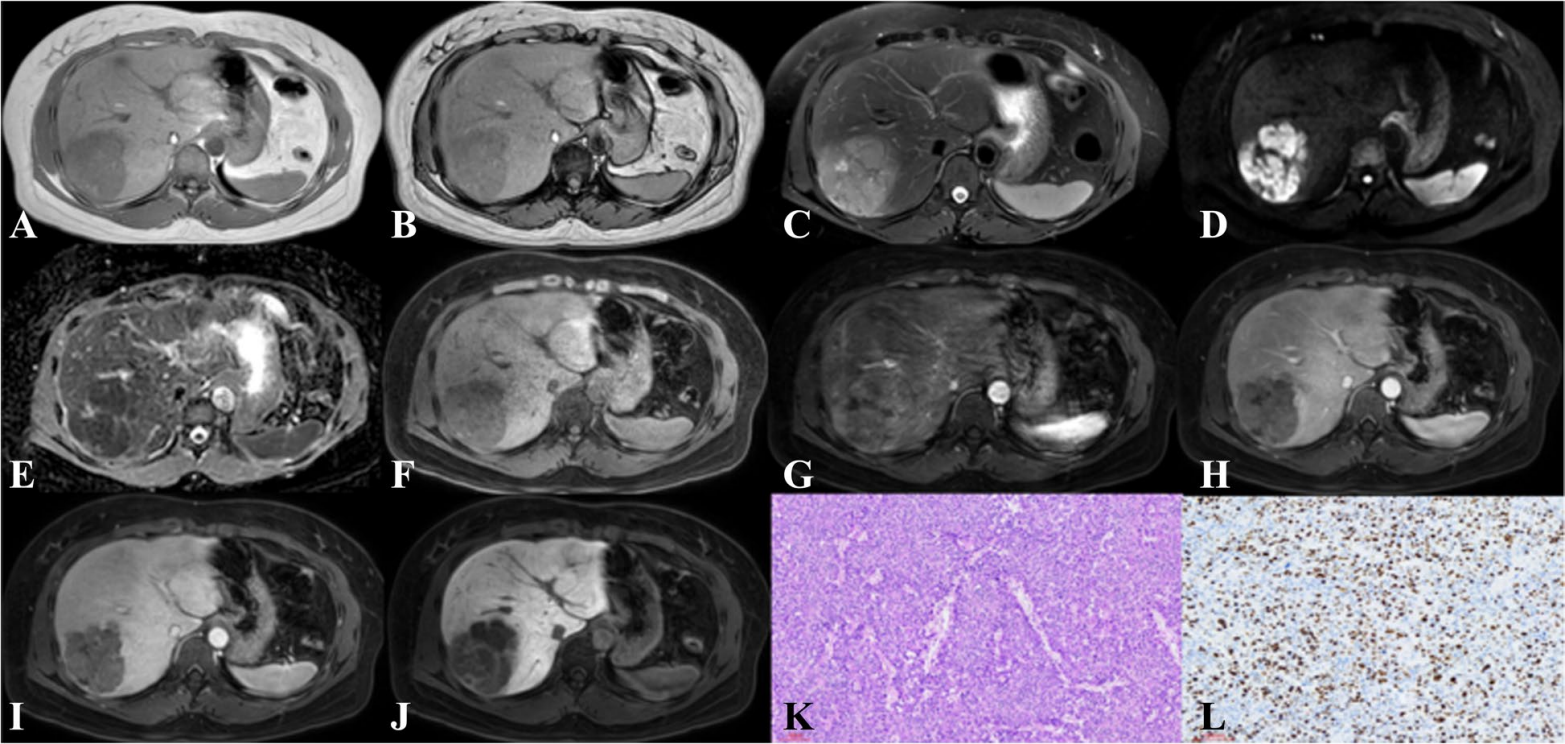
Hepatocellular carcinoma in the right lobe of liver showed heterogenous hypointense with blood products on T1-weighted imaging (**A**, **B**), heterogenous hyperintense on T2-weighted imaging (**C**), hyperintense on DWI (D) while hypointense on apparent diffusion coefficient map (**E**), hypointense on precontrast T1-weighted imaging (**F**), arterial phase hyperenhancement (APHE) on arterial phase (**G**), washout, mosaic architecture and infiltrative appearance on portal venous phase (**H**) and transitional phase (**I**), and apparent hypointense without targetoid appearence on hepatobiliary phase (**J**). **H** Pathology revealing hepatocellular carcinoma (HE × 10). **I** Immunohistochemistry showing high proliferative activity of tumor cells with approximately 50% Ki-67 expression (× 10)

For Ki-67 ≥ 50%, univariate analysis suggested that tumor diameter ≥ 5 cm (*p* = 0.070), presence of nodule-in-nodule architecture (*p* < 0.001), presence of mosaic architecture (*p* < 0.001), presence of blood products in the mass (*p* = 0.039), absence of mild-to-moderate T2 hyperintensity (*p* = 0.023), presence of coronal enhancement (*p* = 0.025), absence of targetoid HBP (*p* = 0.091), presence of infiltrative appearance (*p* = 0.009), *presence* of portal and hepatic vein tumor thrombus (*p* = 0.055), and presence of peritumoral enhancement (*p* = 0.057) were potential differential factors for the prediction of HCC. Subsequently, multivariate logistic analysis was conducted on these potential differential factors. Only the presence of mosaic architecture (OR = 5.507, 95% confidence interval [CI]: 1.036–29.271, *p* = 0.045), presence of infiltrative appearance (OR = 3.129, 95% CI: 1.058–9.258, *p* = 0.039), and absence of targetoid HBP (OR = 0.271, 95% CI: 0.081–0.915, *p* = 0.035) were independent differential factors for prediction of HCC when Ki-67 ≥ 50% (Fig. 3).

### Comparison of diagnostic performance

Table 4 summarizes the corresponding AUCs, 95% CIs, sensitivities, and specificities of the different features and models. The mosaic architecture, infiltrative appearance, and targetoid HBP in the mass exhibited sensitivities of 88.89% (95% CI: 75.90–96.30), 77.78% (95% CI: 62.90–88.80), and 75.56% (95% CI: 60.50–87.10), specificities of 56.58% (95% CI: 44.70–67.90), 46.05% (95% CI: 34.50–57.90), and 39.47% (95% CI: 28.40–51.40) and AUC of 0.727 (95% CI: 0.639–0.804), 0.619 (95% CI: 0.526–0.706), 0.575 (95% CI: 0.482–0.665) for the prediction of the HCC with Ki-67 ≥ 50%, respectively.

**Table 4:** Predictive performance of the model

Appearance	AUC	95%CI	Sensitivity	95%CI	Specificity	95%CI
Mosaic architecture	0.727	0.639, 0.804	88.89	75.9, 96.3	56.58	44.7, 67.9
Infiltrated appearance	0.619	0.526, 0.706	77.78	62.9, 88.8	46.05	34.5, 57.9
Targetoid HBP	0.575	0.482, 0.665	75.56	60.5, 87.1	39.47	28.4, 51.4
Model A	0.781	0.696, 0.851	75.56	60.5, 87.1	77.63	66.6, 86.4
Model B	0.746	0.659, 0.821	88.89	75.9, 96.3	56.58	44.7, 67.9
Model C	0.669	0.578, 0.752	60.00	44.3, 74.3	71.05	59.5, 80.9
Model D	0.776	0.691, 0.847	84.44	70.5, 93.5	63.16	51.3, 73.9

Model A: mosaic architecture and infiltrated appearance

Model B: mosaic architecture and targetoid HBP

Model C: infiltrated appearance and targetoid HBP

Model D: mosaic architecture, infiltrated appearance and targetoid HBP

*Abbreviations*: *AUC* the areas under the receiver operating characteristic curves (AUC), *CI* confidence interval, *HBP* hepatobiliary phase

**Fig. 4:**
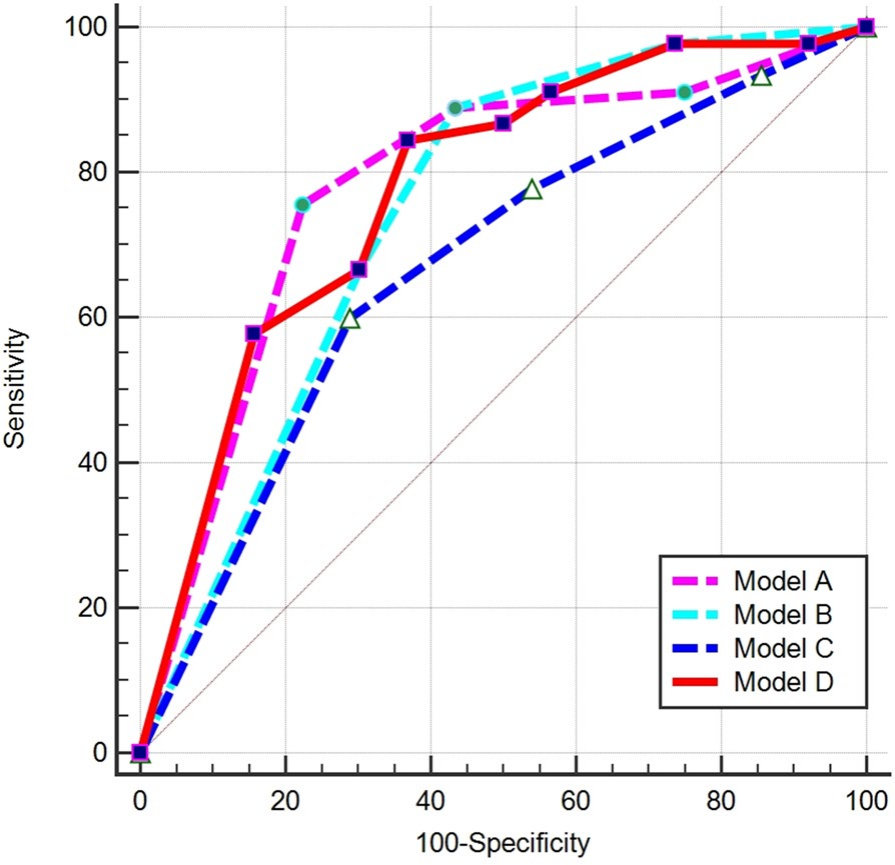
The ROC curve of the models. Model A: mosaic architecture and infiltrated appearance; Model B: mosaic architecture and targetoid hepatobiliary phase; Model C: infiltrated appearance and targetoid HBP; Model D: mosaic architecture, infiltrated appearance and targetoid HBP

Four different logistic regression models were built based on MRI features, including model A (mosaic architecture and infiltrated appearance), model B (mosaic architecture and targetoid HBP), model C (infiltrated appearance and targetoid HBP), and model D (mosaic architecture, infiltrated appearance and targetoid HBP). The ROC curves of the four models are shown in Fig. 4. When the presence of any two features was used to predict HCC with Ki-67 ≥ 50%, the sensitivities were 75.56% (95%CI: 60.50–87.10) in model A, 88.89% (95% CI: 75.90–96.30) in model B, 60.00% (95% CI: 44.30–74.30) in model C with a specificity of 77.63% (95% CI: 66.60–86.40) in model A, 56.58% (95% CI: 44.70–67.90) in model B, 71.05% (95% CI: 59.50–80.90) in model C. The model D based on three predictors yielded a sensitivity, specificity, and AUC of 84.44% (95% CI: 70.50–93.50), 63.16% (95% CI: 51.30–73.90), and 0.776 (95% CI: 0.691–0.847), respectively, for the prediction of HCC when Ki-67 ≥ 50% (Fig. 4). The model D yielded better diagnostic performance than the model C (0.776 vs. 0.669, *p* = 0.002), but a comparable AUC than model A (0.776 vs. 0.781, *p* = 0.855) and model B (0.776 vs. 0.746, *p* = 0.076) (Table 5).

**Table 5:** Comparison of ROCs in predicting models using the Delong test

Model	Z statistic	*P* value
Model D-Model A	0.183	0.855
Model D-Model B	1.777	0.076
Model D-Model C	3.099	0.002
Model C-Model A	2.785	0.006
Model C-Model B	1.587	0.113
Model B-Model A	1.066	0.287

Abbreviations: Model A: mosaic architecture and infiltrated appearance; Model B: mosaic architecture and targetoid HBP; Model C: infiltrated appearance and targetoid HBP; Model D: mosaic architecture, infiltrated appearance and targetoid HBP

## Discussion

Our study showed that the presence of mosaic architecture, infiltrative appearance, and absence of targetoid HBP were independent predictors of Ki-67 (Ki-67 index ≥ 50%) positivity in patients with HCC. A noninvasive multivariable model composed of three LI-RADS features was developed to predict the Ki-67 index in patients with HCC; the model showed good discriminative performance with an AUC of 0.776, and this may be an effective imaging approach for the risk stratification of patients with HCC.

Many studies have confirmed that high Ki-67 expression levels are associated with tumor invasiveness and poor prognoses in patients with HCC [20, 23]. However, there is still no consensus about the precise cut-off value for Ki-67 because values ranging from 5 to 50% are used yet [6, 24–28]. To date, no studies have evaluated the correlation between MRI LI-RADS features and different Ki-67 expression. In the present study, LI-RADS features were compared between the low and high Ki-67 index groups (Ki-67 index ≥ 20% vs. < 20%; Ki-67 index ≥ 30% vs. < 30%; Ki-67 index ≥ 50% vs. < 50%), which demonstrated that there were no LI-RADS features showing statistically significant differences in predicting Ki-67 cut-off values of 20% (Ki-67 index ≥ 20% vs. < 20%) and 30% (Ki-67 index ≥ 30% vs. < 30%). A possible explanation is that although the tumors with different levels of Ki-67 expression may have different components and tissue structures, which may be overlapped with imaging findings in HCC. Thus, more prospective studies with a larger sample size are needed to confirm this result in the future.

In this study, the results also showed that the LI-RADS features including mosaic architecture, infiltrative appearance, and targetoid HBP are sensitive in predicting high Ki-67 expression (Ki-67 index ≥ 50% vs. < 50%) in patients with HCC. Mosaic architecture is a well-known ancillary feature of HCC characterized by random internal nodules or components of different attenuations, intensities, enhancements, sizes, shapes, and separation by fibrous material within tumors [29]. Mosaic architecture may reflect tumor heterogeneity, corresponding to histological variations, including tumor viability, fatty infiltration, necrosis, hemorrhage, cystic degeneration, or copper deposition, suggesting that the internal components of HCCs are complex [30]. It is more common in progressed HCC rather than early HCC [29]. The results of the our study are consistent with the study by Liu [18]. Infiltrative appearance and targetoid HBP are uncommon in HCC and more common in cholangiocarcinoma. Infiltrative appearance was observed in approximately 8%–20% of all HCC cases [31]. Ki-67-positive HCCs have a more infiltrative appearance than conventional Ki-67-negative HCCs. Thus, infiltrative appearance is a key feature of Ki-67-positive HCCs, which may represent true infiltration of tumor cells into the liver parenchyma, and has been associated with more aggressive tumors, metastasis, and short survival times [32, 33]. Targetoid HBP was rarely observed in HCC in our study, especially in Ki-67-positive HCCs. However, it was more frequently observed in CK19-positive HCCs, which suggests the tumor progenitor phenotype [34].

Several studies have evaluated the diagnostic value of the different models for predicting Ki-67 expression [15, 17–19, 35], however, most of the studies are on the basis of radiomics. Wu et al. conducted a radiomics nomogram based on CT features, AFP, and Edmondson grades to predict high Ki-67 expression (≥ 20%) with AUCs of 0.884 and 0.819 in the training and validation groups, respectively [19]. Fan developed a combined model including artery phase Rad-scores and serum AFP levels based on enhanced MRI to predict high Ki-67 expression (≥ 14%) in HCC, which performed better than the artery phase radiomics model in the training (AUC: 0.922 vs. 0.873) and validation cohorts (AUC: 0.863 vs. 0.813) [15]. Undoubtedly, the above previous studies indicated that radiomics was important for predicting Ki-67 expression [19]; however, it requires large sample sizes and is time-consuming. Thus, the present study is the first one to develop a preliminary multivariable model based on LI-RADS features for individualized discrimination of high-level Ki-67 HCCs. The developed model, which included mosaic architecture, infiltrative appearance, and targetoid HBP, was proved to be the best predictive combination with an AUC of 0.776. This model is clinically significant because it is simple and user-friendly, which enables clinicians to implement it.

Our study has several limitations. First, there was the potential for selection bias due to the study being a retrospective, single-center study. Second, the study was limited by the small sample size, and a prospective study with more cases is needed. Third, three non-high-risk patients were included in our study, which may affect the result because LI-RADS version 2018 specifically defined high-risk patients. Finally, a multivariable model was built for the prediction of Ki-67 expression; however, the performance and reproducibility of the model requires further testing using additional methods of external validation due to the limited number of cases.

In conclusion, our study showed that the presence of mosaic, infiltrative appearance, and the absence of targetoid HBP are independent predictors of Ki-67 indexes ≥ 50% in patients with HCC. A noninvasive multivariable model composed of three LI-RADS features was developed to predict the Ki-67 index in patients with HCC, which showed good discriminative performance, with an AUC of 0.776, and may be an effective imaging approach for the risk stratification in patients with HCC.

## Data Availability

The datasets used and analysed during the current study are available from the corresponding author on reasonable request.
